# Supplementary material for: Comparative multiomics analysis of cell physiological state after culture in a basket bioreactor
Source: Sci Rep. 2022 Nov 23;12:20161. doi: 10.1038/s41598-022-24687-4 (PMC9686226; doi:10.1038/s41598-022-24687-4)
Supplement: Supplementary file 1 — Supplementary Information 1. [file 41598_2022_24687_MOESM1_ESM.zip › raw data/Proteomic raw data/6.KEGGenrichment/EG.vs.CG/ALL/pathway/src/04910.html]

map04910


- K06269, K07189, K17453

- Down regulated proteins


A0A0D9RU34(Description:CBM21 domain-containing protein;Gene:PPP1R3E;log2FC:-inf)

- K00693

- Down regulated proteins

A0A0D9R6H1(Description:Glycogen [starch] synthase;Gene:GYS2;log2FC:-1.1230)

A0A0D9S336(Description:Glycogen [starch] synthase;Gene:GYS1;log2FC:-1.6625)

- K03259

- Up regulated proteins


A0A0D9R2B7(Description:Uncharacterized protein;Gene:EIF4E2;log2FC:1.8125)

- K02158

- Up regulated proteins

A0A0D9R491(Description:Uncharacterized protein;Gene:BAD;log2FC:inf)

- K04707

- Up regulated proteins

A0A0D9R1V2(Description:E3 ubiquitin-protein ligase CBL;Gene:CBLB;log2FC:1.1276)

Close
